# OmniEdit: A unified CRISPR/Cas9 platform for precise genome engineering and strain optimization of the *Cordyceps militaris* cell factory

**DOI:** 10.1016/j.synbio.2026.04.015

**Published:** 2026-05-17

**Authors:** Mengqian Liu, Sijin Chen, Jingting Wang, Guoliang Meng, Caihong Dong

**Affiliations:** a State Key Laboratory of Microbial Diversity and Innovative Utilization, Institute of Microbiology, Chinese Academy of Sciences, Beijing, 100101, China; b School of Pharmacy, Zunyi Medical University, Zunyi, 563000, China; c Shandong Provincial Key Laboratory of Applied Mycology, College of Life Sciences, Qingdao Agricultural University, Qingdao, 266109, China

**Keywords:** Point mutation, Fluorescent protein tagging, Large fragment deletion, uORF editing, One-step dual-gene knockout

## Abstract

*Cordyceps militaris* serves as a critical microbial cell factory for high-value bioactive compounds; however, the scarcity of versatile and sophisticated genome-editing toolsets significantly restricts its systematic metabolic engineering. This study aimed to develop OmniEdit, a unified and highly efficient CRISPR/Cas9-based platform, to streamline diverse and complex genetic modifications for strain engineering. We integrated multiple editing modalities into a standardized workflow utilizing an AMA1-based CRISPR/Cas9 system combined with customized homologous donor templates. The platform's robustness was systematically validated through five distinct engineering tasks delivered via PEG-mediated transformation, including precise point mutation, *in situ* fluorescent protein tagging, large biosynthetic gene cluster (BGC) deletion, regulatory upstream open reading frame (uORF) disruption, and one-step multiplexed gene knockout. OmniEdit achieved the first CRISPR-based precise point mutation (∼4–5% efficiency) in this fungus. It enabled the surgical excision of an entire ∼26 kb BGC (∼10% efficiency) and efficient one-step dual-gene knockout (∼30% efficiency). Furthermore, targeted uORF editing was implemented to modulate translational efficiency, alongside flexible protein tagging for subcellular analysis. By overcoming technical bottlenecks, OmniEdit provides a standardized, powerful toolkit for functional genomics and the systematic enhancement of *C. militaris* as a high-performance bioproduction platform.

## Highlights

• OmniEdit, a unified CRISPR/Cas9 platform, was developed for *Cordyceps militaris*.
• First reported point mutation and uORF editing in mushroom-forming fungi.
• Precise excision of a 26 kb gene cluster and one-step multiplexed knockout achieved.
• A standardized toolkit for building high-performance microbial cell factories.

## Graphical abstract

**Figure:**
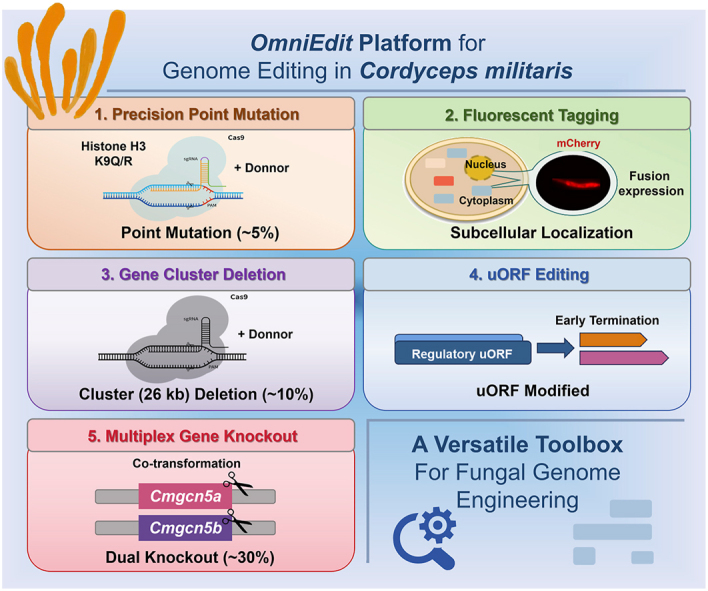

## 1 Introduction

*Cordyceps militaris*, a prominent ascomycete fungus, has gained global recognition not only as a traditional medicinal mushroom but also as an important microbial cell factory [1]. It is the primary industrial source of bioactive secondary metabolites such as cordycepin and pentostatin [2], which possess significant pharmacological properties [3]. With the rapid development of biochemical engineering, there is a growing demand to systematically optimize *C. militaris* strains for enhanced titer, purity, and safety. However, the lack of versatile, high-efficiency genetic manipulation tools has long remained the primary bottlenecks in its metabolic and functional genomic research.

CRISPR/Cas9-based genome editing technologies in *C. militaris* have advanced rapidly in recent years. An early implementation employing the Cas9 DNA endonuclease, RNA presynthesized in vitro, and a single-strand DNA template successfully achieved site-specific deletions and insertions [4]. Subsequent ribonucleoprotein (RNP) delivery further reduced genomic integration risks but suffered from labor-intensive screening due to the lack of selectable marker [5]. A significant advance came with an AMA1-based autonomous CRISPR/Cas9 system, which improved knockout efficiency using a *tRNA*Pro promoter and enabled scarless editing via plasmid loss after transformation [6]. Shortly after, Chen et al. [7] reported a scalable system deleting a 10 kb biosynthetic cluster. We subsequently developed an AMA1-based safe-harbor CRISPR/Cas9 system for stable gene integration [8]. These knock-in and knock-out systems support the scarless editing and have been successfully applied not only in functional gene characterization [9,10], but also to strain improvement, including enhanced disease resistance [8], improved strain stability [11], and elimination of the mycotoxin ustiloxin biosynthesis [12]. Despite these advances, current CRISPR/Cas9 tools remain fragmented and largely task-specific, highlighting the need for a unified and versatile genome-editing platform capable of supporting diverse genetic operations within a single standardized framework.

Precise point mutation is essential for functional genomics, yet remains challenging in fungi due to reliance on inefficient homologous recombination [13]. In contrast, plant systems have benefited from the development of cytosine base editors [14], adenine base editors [15], glycosylase base editors [16] and prime editing, enabling nucleotide substitutions with high specificity [17,18]. However, these advanced editing systems have not yet been adapted for fungal research, highlighting a major technological gap.

Fluorescent protein tagging is another fundamental genetic tool, enabling visualization of protein subcellular localization and downstream biochemical analyses [19]. In fungi, conventional tagging strategies rely on spontaneous double-strand break (DSB) repair and random homologous recombination, resulting in low efficiency [20]. Similarly, functional interrogation of secondary metabolism often requires deletion of entire biosynthetic gene clusters (BGCs). While CRISPR/Cas9-mediated cluster deletion using dual sgRNAs has been demonstrated in cell lines [21], achieving precise, predictable removal of large genomic regions remains technically demanding.

Beyond transcriptional regulation, fine-tuning gene expression at the translational level through upstream open reading frame (uORF) editing has emerged as a powerful strategy in plants, enabling quantitative modulation of protein output without altering coding sequences [22,23]. However, uORF editing remains largely unexplored in mushroom-forming fungi. Likewise, multiplex CRISPR/Cas9 editing is increasingly essential for dissecting genetic redundancy and complex traits, yet current fungal systems suffer from limited efficiency and scalability, particularly when relying on tandem sgRNA arrays or co-transformation strategies [24].

The lack of an integrated and versatile genome-editing platform has limited both fundamental and applied research in mushroom-forming fungi. In this study, we developed OmniEdit (Omnipotent Editor), a comprehensive and versatile CRISPR/Cas9 platform designed to streamline genome engineering for *C. militaris*. By integrating an AMA1-based system with tailored donor templates, OmniEdit offers a workflow for diverse engineering requirements. Specifically, we achieved the first precise point mutation alongside the deletion of a ∼26 kb BGC, demonstrating its capacity for both residue-specific analysis and large-scale genomic streamlining. The platform's versatility is further showcased through *in situ* protein tagging, targeted uORF disruption for translational control, and one-step multiplexed gene knockout. By systematically addressing these technical bottlenecks, OmniEdit provides a robust foundation for the precise enhancement of *C. militaris* as a high-performance bioproduction platform, aligning with the goals of modern bioprocess optimization.

## 2 Materials and methods

### 2.1 Strains and culture conditions

The wild-type (WT) *C. militaris* strain CGMCC 3.16323 was maintained on potato dextrose agar (PDA) at 20 °C. *Escherichia coli* DH5α chemically competent cells, which were used for plasmid construction, were purchased from Genesand Biotech Co., Ltd. (Beijing, China) and were stored at −80 °C. *E. coli* strains were cultured in Luria-Bertani medium supplemented with ampicillin (100 μg/mL) for selection.

### 2.2 Plasmid construction

The parental CRISPR/Cas9 vector pAMA1-Cas9-hyg, which expressed a codon-optimized Cas9 and a hygromycin B resistance marker (*hph*), was used as the backbone for all genome-editing constructs [6]. All sgRNAs were designed using the Eukaryotic Pathogen CRISPR guide RNA/DNA Design Tool (http://grna.ctegd.uga.edu/) with default parameters, targeting specific nucleotide sequences in the *C. militaris* CM01 reference genome (GCF_000225605.1) [25]. A DNA Assembly Mix Plus kit (LABLEAD Trading Co., Ltd., Beijing, China) was employed for the assembly of all DNA fragments into the vector.

To introduce the H3K9R (AAG-to-AGG) or H3K9Q (AAG-to-CAG) substitution into the *CmH3* gene (*CCM_07449*), sgRNAs were designed proximal to the target site. The selected sgRNA (sgRNA1, located 112 bp from the mutation site) was inserted into pAMA1-Cas9-hyg to generate the intermediate vector. An HDR donor containing the desired point mutation and ∼1 kb homology arms was generated by PCR amplification. Notably, a 28 bp segment immediately downstream of the *CmH3* stop codon was omitted from the donor to facilitate screening. The HDR fragments were cloned into the intermediate vector, resulting in the final editing plasmid. A second vector harboring a proximal sgRNA2 located 56 bp from the mutation site was constructed using the same strategy for efficiency comparison.

For *in situ* C-terminal tagging of *Cmgcn5a* with *mCherry* at its native locus, an sgRNA targeting a site 230 bp downstream of the stop codon was used. The HDR donor was designed to remove the native stop codon and replace it with an in-frame sequence encoding a flexible linker (GSGGGGSGGGGS) followed by the *mCherry* coding sequence. To prevent re-cleavage by Cas9, the genomic region spanning from the sgRNA target site to the original stop codon was deleted in the HDR donor. For safe-harbor integration, a fusion construct consisting of the *Cmgcn5a* coding sequence (without a stop codon), the same linker, *mCherry*, and the strong constitutive *CmgpdA* promoter was cloned into an HDR donor targeting a predefined genomic safe harbor site.

For deletion of the ∼26 kb ustiloxin biosynthetic gene cluster (BGC), sgRNAs targeting the terminal genes *CCM_02054* and *CCM_02066* were designed, and their expression cassettes were cloned into a single vector. Homology arms of 800 bp (upstream) and 828 bp (downstream), corresponding to genomic sequences immediately flanking the BGC, were amplified and assembled into the same vector as the donor. Control vectors containing only one sgRNA (targeting either *CCM_02054* or *CCM_02066*) in combination with the same donor were also constructed.

Putative uORFs were predicted using NCBI ORF finder (https://www.ncbi.nlm.nih.gov/orffinder/Paste/) and uORF light (http://uorflight.whu.edu.cn). To disrupt uORFs in the 5′UTR of *CCM_08965*, sgRNAs were designed to target sequences adjacent to the start codons of uORF1 and uORF3. Corresponding CRISPR/Cas9 editing vectors were assembled by cloning the sgRNA expression cassette into the pAMA1-Cas9-hyg backbone.

To generate double-gene knockout vectors for *Cmgcn5a* and *Cmgcn5b*, a one-step multiplexed knockout strategy was employed. For each gene, a specific sgRNA expression cassette (*CmtRNApro*-sgRNA-T6) was first cloned into the parental vector pAMA1-Cas9-hyg, generating intermediate constructs (pAMA1-Cas9-hyg-*Cmgcn5a* and pAMA1-Cas9-hyg-*Cmgcn5b*). Subsequently, approximately 1 kb of upstream (left arm) and downstream (right arm) homologous sequences flanking the target gene open reading frame were amplified from WT genomic DNA and assembled into the corresponding intermediate vector via a one-step cloning strategy. This yielded the final knockout constructs: pAMA1-Cas9-sgRNA_*Cmgcn5a*-ko_ and pAMA1-Cas9-sgRNA_*Cmgcn5b-ko*_.

### 2.3 Protoplast-mediated transformation and screening

Protoplast preparation and polyethylene glycol (PEG)-mediated transformation were performed according to a previously described procedure [6]. *C. militaris* strain was cultured in 100 mL of PPDB (potato dextrose broth supplied with 1% peptone) at 20 °C for 4 d under static condition. For protoplast generation, 0.2 g of wet mycelia were digested in 1 mL of 2% lywallzyme (dissolved in 0.8 mol/L KCl, pH 6.5; Guangdong Culture Collection Center, Guangzhou, China) at 32 °C with gentle shaking (90 rpm) for 3 h. The resulting protoplasts were filtered through four layers of lens paper, washed twice with 0.8 mol/L KCl by centrifugation at 3000 rpm for 10 min at room temperature, and resuspended in STC buffer (1 M sorbitol, 10 mM Tris-HCl, 25 mM CaCl_2_, pH 7.5) to a final density of 3 × 10^8^ cells/mL.

For transformation, 2 μg of plasmid was added to 100 μL of protoplast suspension. After incubation on ice for 5 min, 50 μL of PEG buffer (25% PEG, 10 mM Tris-HCl, 25 mM CaCl_2_, pH 7.5) was added, followed by incubation on ice for 30 min. Subsequently, 0.5 mL of PEG buffer was added and incubated at 28 °C for 20 min, then diluted with 1 mL of STC buffer. Cells were harvested by centrifugation at 3000 rpm for 10 min at room temperature, resuspended in 200–400 μL of STC buffer, and plated on PPDA supplemented with 1 M mannitol and 500 μg/mL hygromycin at 25 °C. Colonies were obtained after 4–7 d and transferred to fresh selection plates (PPDA with 500 μg/mL hygromycin).

For gene knockout mutants, screening was conducted using primers located outside the homology arms and within the deleted region. For point mutations and large genomic deletions, a two-step PCR screening strategy was employed. The first PCR was performed to confirm the loss of the WT locus, and the second PCR used primers flanking the edited region to verify precise editing. The specific locations of all verification primers were shown in the corresponding figures, and all primer sequences were provided in Table S1.

### 2.4 Prediction of transcription start sites and validation by RT-PCR

*In silico* promoter analysis of the 1865 bp region upstream of the *CCM_08965* translation start codon (ATG) was conducted using Promoter 2.0 (https://services.healthtech.dtu.dk/services/Promoter-2.0/) and the BDGP network predictor (https://fruitfly.org/seq_tools/promoter.html) and putative transcription start sites (TSSs) were identified.

To experimental validate transcript initiation from these predicted TSSs, reverse-transcription PCR (RT-PCR) analysis was performed using three forward primers positioned −79 bp (F1), −342 bp (F2), and −609 bp (F3) relative to the ATG, paired with a common reverse primer (R) situated within the second exon of *CCM_08965*. Total RNA was extracted from mycelia of WT *C. militaris* strain using the E.Z.N.A.® Total RNA Kit I (Omega Bio-tek Inc., Norcross, GA, USA). First-strand cDNA was synthesized using the HiScript III RT SuperMix for qPCR (+gDNA wiper) kit (Vazyme Biotech Co., Ltd., Beijing, China). PCR amplification was performed using both cDNA and genomic DNA (gDNA) as templates. cDNA-specific PCR products were gel-purified and sequenced to confirm correct splicing of the first intron.

### 2.5 Fluorescence microscopy

Mycelia were stained using the Calcofluor White (CFW) Fungal Fluorescence Stain Kit (Solarbio Science & Technology Co., Ltd., Beijing, China) and DAPI (2-4-Amidinophenyl-6-indolecarbamidine dihydrochloride) Staining Solution (Beyotime Biotech Inc., Beijing, China) at room temperature. CFW staining was performed according to the manufacturer's instructions. DAPI was dissolved in 0.01 mol/L PBS buffer (pH 7.0) to a final concentration of 20 μg/mL. For staining, 5 μL of the staining solution was applied to the coverslips bearing mycelia, followed by fluorescence microscopy observation. Fluorescence signals were subsequently observed and imaged using a Zeiss IMAGER A2 fluorescence microscope (Göttingen, Germany).

### 2.6 Sample preparation and HPLC analysis of GABA

The extraction and quantification of GABA were performed with reference to a previously described method, with appropriate modifications [26]. Exactly 50 mg of mycelial or fruiting body powder was accurately weighed into a 10 mL centrifuge tube. Ultrapure water was added to achieve a solid-to-liquid ratio of 1:100, and the mixture was thoroughly mixed. Samples were extracted at 65 °C for 1 h, followed by centrifugation at 12,000 rpm for 10 min. The supernatant was filtered through a 0.22 μm nylon membrane. For derivatization, 80 μL of GABA standard or samples solution was mixed with 180 μL of ultrapure water, 160 μL of 0.1 mol/L borate buffer (pH 8.6), 240 μL of acetonitrile, and 160 μL of 4 mmol/L FMOC-Cl (dissolved in acetonitrile). The mixture was vortexed and incubated at 40 °C for 5 min. The reaction solution was then filtered through a 0.22 μm nylon membrane prior to HPLC analysis.

GABA content was analyzed by reversed-phase HPLC using a Shimadzu LC-20A Liquid Chromatographs Series (Shimadzu Corporation, Kyoto, Japan) equipped with a parallel dual-plunger pump, SPD-20A detector, SIL-20A autosampler and LC solution for data analysis. The injection volume was 20 μL, the detection wavelength was 265 nm, and the run time was 25 min. Separation was carried out under isocratic conditions using a mobile phase consisting of 30% mobile phase A [50 mmol/L sodium acetate buffer (pH 4.8)-methanol-acetonitrile-tetrahydrofuran (82:8.5:8.5:1, V/V)] and 70% mobile phase B [50 mmol/L sodium acetate buffer (pH 4.8)-methanol-acetonitrile-tetrahydrofuran (22:38.5:38.5:1, V/V)] at a flow rate of 1.0 mL/min and a column temperature of 40 °C.

### 2.7 Data analysis and statistics

Genome-editing efficiency was calculated as the percentage of hygromycin-resistant transformants that yielded a positive diagnostic PCR band, confirmed by sequencing, relative to the total number of transformants screened. All experiments were performed with at least three independent biological replicates. Data were presented as mean ± standard deviation (SD). All statistical analyses were performed using GraphPad Prism 8.0 (GraphPad Software Inc., CA, USA) and IBM SPSS 16.0 (SPSS Inc., IL, USA). Student's *t*-test was used to determine statistical significance between two groups, while one-way analysis of variance (ANOVA) was employed for comparisons involving more than two groups, followed by multiple range tests.

## 3 Results

### 3.1 Precision CRISPR/Cas9-mediated point mutations

**Fig. 1:**
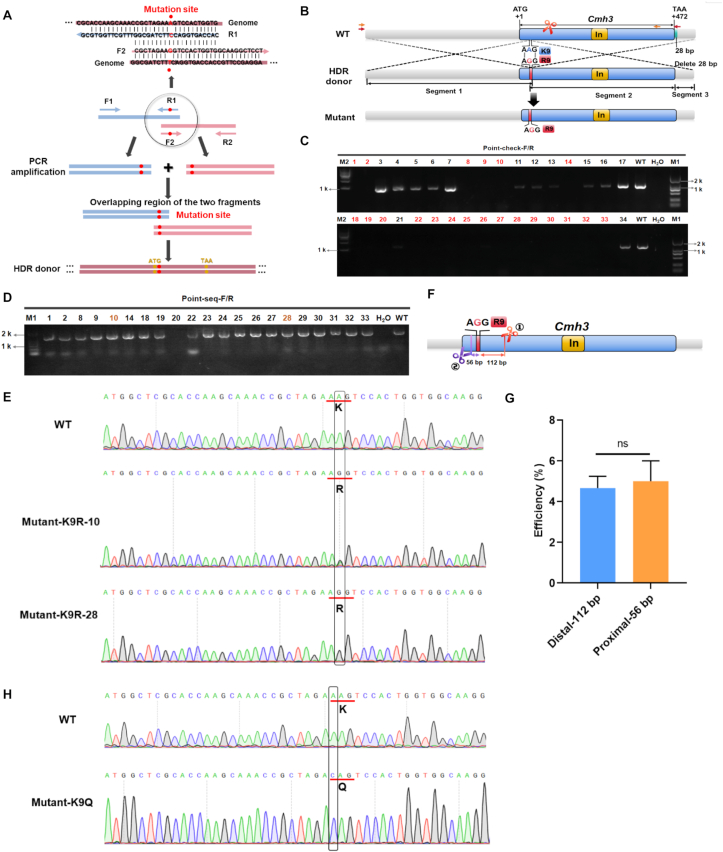
**OmniEdit-mediated point mutation strategy and validation in *Cordyceps militaris.*** **A:** Strategy for generating the HDR donor via PCR amplification. The upstream fragment was amplified using primers F1/R1, and the downstream fragment using primers F2/R2. The overlapping region between the two fragments contained the intended point mutation. **B:** Schematic overview of the point mutation editing strategy. The scissors icon indicated the sgRNA target site. Segments 1, 2, and 3 represented the three fragments amplified for vector construction. Segment 3 was intentionally amplified without the 28 bp region immediately downstream of the stop codon (highlighted in green) to facilitate subsequent screening. Primers used for primary screening (point-check-F/R) were marked in red, and primers used for secondary sequencing validation were shown in orange-yellow. “In” denoted gene introns. **C:** Primary PCR screening of transformants. Samples that failed to yield an amplification product were labeled with red numbers and were subjected to second PCR validation. M1: 2 kb DNA marker; M2: 10 kb DNA marker. WT: wild-type strain CGMCC 3.16323. **D:** Secondary PCR verification of selected transformants. PCR products were subsequently subjected to Sanger sequencing. The numbers corresponded to those in Figure C. **E:** Sequence alignment of transformants carrying the lysine-to-arginine (K9R) mutation. The boxed region indicated the nucleotide substitution site, and the red underline indicated the corresponding codon change. Mutant-K9R-10 showed a heterozygous genotype, Mutant-K9R-28 was homozygous, and WT represented the wild-type control. **F:** Location of sgRNAs relative to the mutation site. The original distal sgRNA1 (located 112 bp from the target site) was labeled in red, and the proximal sgRNA2 (located 56 bp from the target site) was marked in purple. **G:** Editing efficiencies achieved using different sgRNAs. Data represented three biological replicates. Statistical significance was assessed by Student's *t*-test; “ns” indicated no significant difference. **H:** Sequence alignment of transformants with lysine-to-glutamine (K9Q) mutation. The boxed region indicated the nucleotide substitution site, and the red underline marked the resulting codon change.

To investigate the role of histone H3 lysine 9 acetylation, a lysine-to-arginine (K9R) point mutation was introduced into the *CmH3* gene (*CCM_07449*) of *C. militaris*. This substitution preserved the positive charge of the residue while abolishing its acetylation potential, thereby mimicking a constitutively non-acetylatable state [27]. An sgRNA1 located 112 bp from the target nucleotide was selected for genome editing, and an HDR donor harboring the AAG-to-AGG substitution was constructed (Fig. 1A). To facilitate efficient screening, a 28 bp sequence immediately downstream of the *CmH3* stop codon was intentionally omitted from the donor (Fig. 1A and B). Following PEG-mediated transformation, transformants were subjected to a two-step PCR screening strategy. Colonies lacking the expected amplicon corresponding to the omitted downstream region were selected to secondary validation (Fig. 1C). Sanger sequencing confirmed precise incorporation of the intended AAG-to-AGG point mutation, corresponding to the lysine-to-arginine (K-to-R) substitution, without any additional sequence alterations (Fig. 1D and E). Across independent experiments, the editing efficiency was stable, with an average success rate of approximately 5% (Fig. 1F and G). One transformant exhibited heterozygosity at the target site (Fig. 1E), likely resulting from mixed protoplast regeneration.

Because Cas9-mediated cleavage occurs 3′ bp upstream of the PAM sequence, reducing the distance between the DSB and the intended edit has been associated with increased HDR efficiency in some systems [28]. To assess whether cleavage proximity influenced editing efficiency in *C. militaris*, a second sgRNA2 (proximal sgRNA) positioned 56 bp from the mutation site was designed and evaluated using the same experimental framework (Fig. 1F and G). However, no statistically significant improvement in editing efficiency was observed relative to the distal sgRNA (Fig. 1G).

To further assess the generalizability of this precision-editing strategy, a lysine-to-glutamine (K-to-Q) mutation was also introduced at the same residue to mimic a constitutively acetylated state of CmH3 (Fig. 1H and S1). Using an identical design, transformation, and screening pipeline, K9Q mutants were successfully obtained with a comparable efficiency of approximately 4%. These results demonstrate that the OmniEdit enables precise, site-specific point mutations in *C. militaris.*

### 3.2 *In situ* and safe-harbor-driven fluorescent tagging of CmGCN5a

**Fig. 2:**
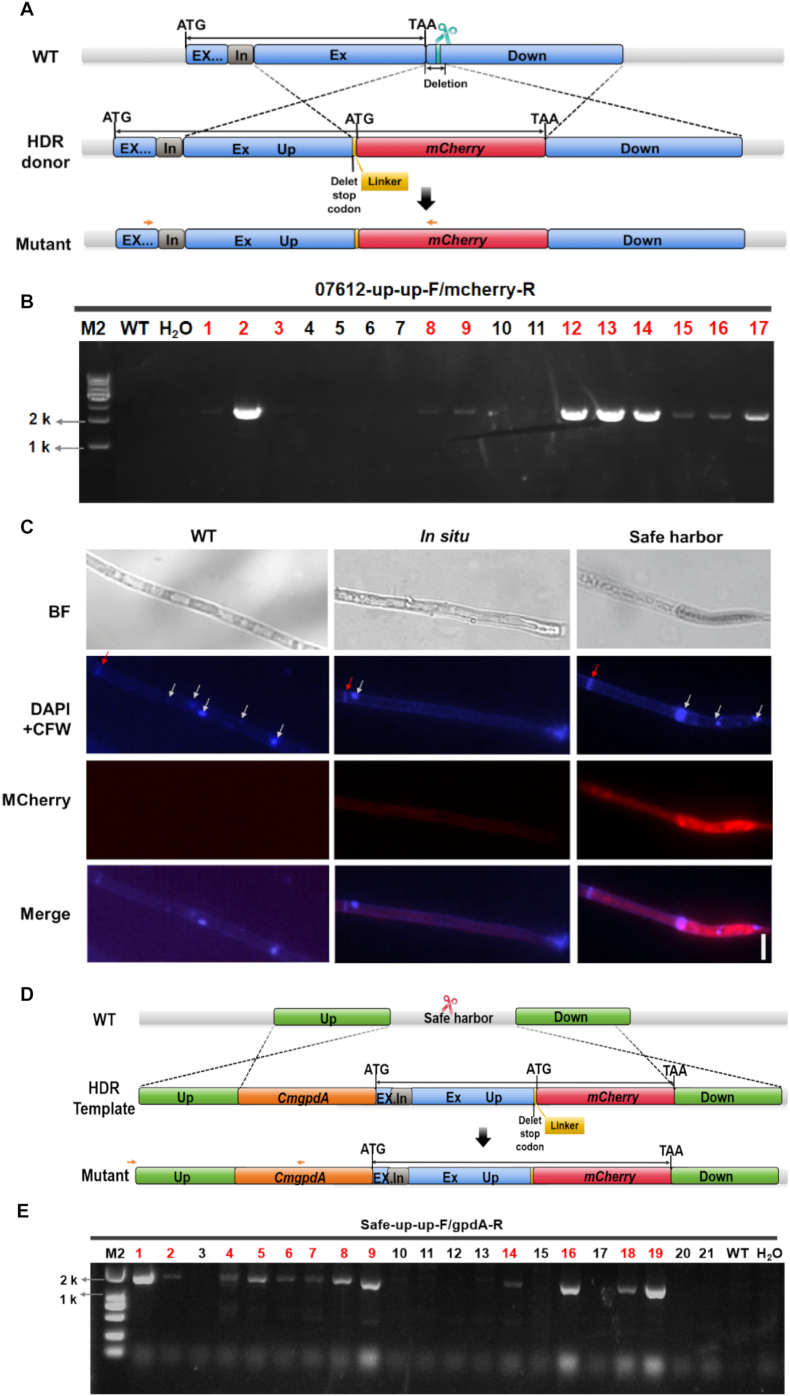
**OmniEdit-mediated *in situ* and safe-harbor-driven fluorescent tagging of CmGCN5a.** **A:** Strategy for *in situ* C-terminal fusion of CmGCN5a with mCherry protein. The sgRNA target site (indicated by green scissors) was designed 230 bp downstream of the *Cmgcn5*a stop codon. Ex: exon; In: intron; Up: upstream homology arm; Down: downstream homology arm. The native stop codon in the final exon was removed and replaced with a sequence encoding a flexible linker (GSGGGGSGGGGS) followed by the *mCherry*. The genomic region between the sgRNA target site and the original stop codon was deleted in the donor to prevent repeated Cas9 cleavage. Orange arrows indicated primers used for diagnostic PCR verification. **B:** PCR verification of *in situ*-tagged transformants. Transformants yielding the expected amplicon (marked with red labels) were identified as positive. M2: 10 kb DNA marker. WT: Wild-type strain CGMCC 3.16323. **C:** Fluorescence microscopy of CmGCN5a–mCherry fusion proteins by *in situ* tagging and safe-harbor promoter-driven expression. Scale bar: 10 μm. BF: bright field; DAPI + CFW: staining with DAPI and Calcofluor White; mCherry: red fluorescent protein signal; Merge: merged fluorescence channels. Nuclei and septa were indicated by white and red arrows, respectively. **D:** Strategy for safe-harbor integration of the *Cmgcn5a-mCherry* fusion driven by a strong constitutive promoter. The sgRNA target site (indicated by red scissors) was designed within the predefined genomic safe-harbor locus. Ex, exon; In, intron; Up, upstream homology arm; Down, downstream homology arm. Orange arrows indicated verification primers. **E:** PCR verification of safe-harbor integrants. Transformants yielding the expected amplicon (marked with red labels) were identified as positive. M2: 10 kb DNA marker. WT: Wild-type strain CGMCC 3.16323. H_2_O, no-template control.

To investigate the subcellular localization of CmGCN5a protein, two distinct CRISPR/Cas9-mediated strategies were employed to generate C-terminal mCherry fusion proteins: *in situ* tagging at the native locus and integration of a fusion construct into a predefined genomic safe-harbor site (Fig. 2).

For *in situ* tagging, an sgRNA targeting a site 230 bp downstream of the *Cmgcn5a* stop codon and an HDR donor were employed (Fig. 2A). Correct integration at the native locus was confirmed by diagnostic PCR using the indicated primer pairs (Fig. 2A and B, orange arrows). Fluorescence microscopy revealed detectable but weak mCherry signals in the resulting strains (Fig. 2C), consistent with limited expression driven by the endogenous *Cmgcn5a* promoter.

To achieve stronger and constitutive fluorescence signals, a *Cmgcn5a*-*mCherry* fusion cassette driven by the strong constitutive *CmgpdA* promoter was integrated into a predefined genomic safe harbor locus [8] (Fig. 2D). Successful integration was verified by PCR screening using primers flanking the insertion site (Fig. 2E). In contrast to the *in situ*-tagged strains, safe-harbor integrants exhibited robust mCherry fluorescence, predominantly localized to the nucleus, with additional signal detected in the cytoplasm (Fig. 2C). These observations confirmed the predominant nuclear localization of CmGCN5a and demonstrated the enhanced signal intensity achieved through safe-harbor-driven expression. It was indicated that the OmniEdit platform enabled flexible fluorescent protein tagging in *C. militaris*, allowing either preservation of the native regulatory context through *in situ* tagging or enhanced signal intensity via safe-harbor-driven expression.

### 3.3 Targeted deletion of large biosynthetic gene clusters (BGCs)

**Fig. 3:**
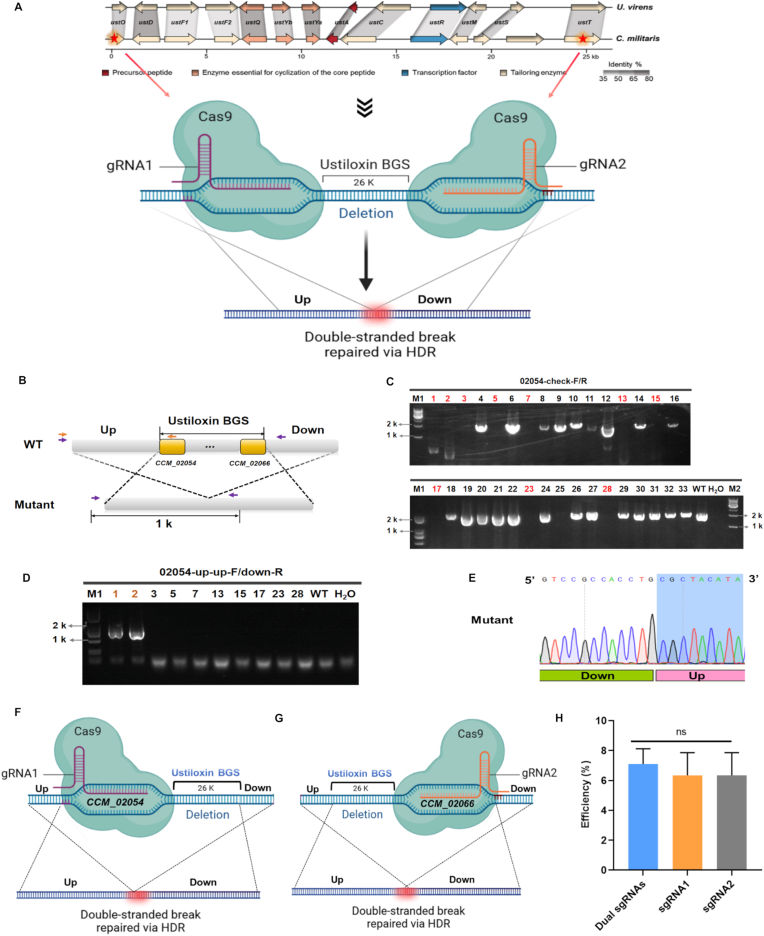
**OmniEdit-mediated deletion of a large biosynthetic gene cluster.** **A:** Schematic overview of the dual-sgRNA strategy used for deletion of the ustiloxin BGC. The positions of sgRNAs target sites were indicated by stars. The upstream (up) and downstream (down) homology arms flanking the BGC were labeled. **B:** Primer design for the two-step PCR screening strategy. Primers used for the primary and secondary screenings were marked in orange and purple, respectively. **C:** Primary PCR screening of transformants. Transformants that failed to yield the expected amplicon (marked with red labels) were selected for secondary validation. M1: 2 kb DNA marker; M2: 10 kb DNA marker. WT: Wild-type strain CGMCC 3.16323. **D:** Secondary PCR verification of candidate transformants. The resulting PCR products were subsequently subjected to Sanger sequencing. **E:** Representative Sanger sequencing chromatogram of a knockout mutant, confirming precise deletion of the entire BGC and direct junction of the upstream and downstream homology arms. **F:** Knockout strategy employing a single sgRNA targeting *CCM_02054* (sgRNA1). **G:** Knockout strategy employing a single sgRNA targeting *CCM_02066* (sgRNA2). **H:** Comparison of knockout efficiencies between single-sgRNA and dual-sgRNA strategies. Data were presented as mean ± SD from three biological replicates. Statistical significance was assessed by one-way ANOVA; “ns,” indicated no significant difference.

Efficient deletion of large genomic segments, particularly entire BGCs, has remained a significant challenge in fungal functional genomics. To address this, a CRISPR/Cas9-mediated OmniEdit strategy was applied to enable precise large-scale genomic deletions in the edible fungus *C. militaris*. The effectiveness of this approach was evaluated by targeting complete removal of the ∼26 kb ustiloxin BGC. Cas9 cleavage was directed to the terminal genes *CCM_02054* and *CCM_02066* (Fig. 3A). Following PEG-mediated transformation, transformants were screened using a two-step PCR strategy to identify strains carrying the expected cluster deletion (Fig. 3B). Primary PCR screening was performed using primers spanning the 5’ flanking region and the *CCM_02054* coding sequence. Transformants lacking the corresponding amplicon were selected for secondary validation (Fig. 3C). Second PCR employed primers flanking the entire deletion junction and yielded an expected ∼1 kb product in candidate strain (Fig. 3D). Sanger sequencing of these PCR products confirmed precise deletion of the entire gene cluster and accurate ligation of the upstream and downstream homology arms (Fig. 3E). Across independent experiments, the deletion efficiency was reproducible, with an average success rate of approximately 10%. Sequence analysis revealed that all validated knockout mutants arose exclusively through HDR, despite the use of two sgRNAs designed to generate dual DSBs at both boundaries of the locus (Fig. 3A and E). This observation suggested that, in the presence of an exogenous donor, HDR was preferentially utilized during successful large-fragment deletion events.

To further examine whether dual sgRNAs were required for efficient cluster deletion, additional vectors containing only a single sgRNA targeting either *CCM_02054* or *CCM_02066*, in combination with the same donor, were constructed and evaluated (Fig. 3F and G). Transformation with these single-sgRNA vectors successfully generated complete BGC deletions (Fig. S2), with efficiencies comparable to those obtained using the dual-sgRNA strategy (Fig. 3H). These results demonstrated that a single Cas9-induced DSB, when coupled with appropriately designed homology arms, was sufficient to drive precise deletion of a large (∼26 kb) genomic segments in *C. militaris*. Furthermore, homology arms of ∼800 bp were adequate to support recombination-mediated removal of large gene clusters in this organism.

Overall, the OmniEdit platform enabled efficient and precise CRISPR/Cas9-mediated large-scale genomic deletions in *C. militaris*.

### 3.4 Targeted editing of 5′ UTR-uORFs

**Fig. 4:**
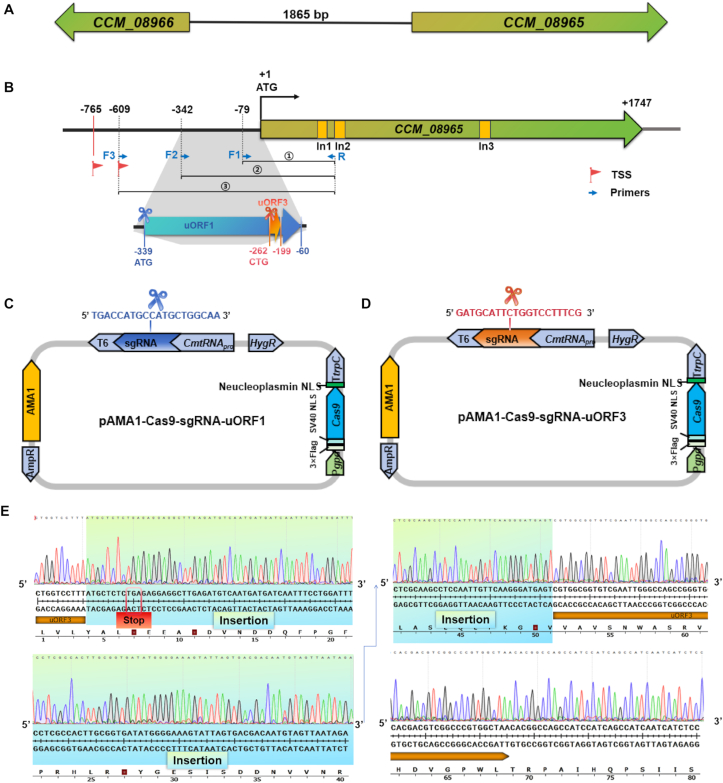
**Target editing of uORF in *CCM_08965.*** **A:** Schematic representation of the genomic organization and transcriptional direction of *CCM_08965* and *CCM_08966*. **B:** Analysis of the promoter region and schematic overview of predicted uORFs of *CCM_08965*. Two predicted transcription start sites (TSS) for *CCM_08965* were identified at −765 (Promoter 2.0) and −609 (BDGP) relative to the ATG. All primers were indicated by blue arrows. Amplified products (numbered 1–3 with circles) and introns of different lengths (labeled as “In”) were marked. uORF1 was shown in blue and uORF3 in orange. Scissors in the corresponding colors indicated sgRNA target sites. Detailed sequences were provided in Fig. S4 C**:** Schematic representation of the editing vector targeting uORF1. The blue nucleotide sequence at the scissor icon indicated the sgRNA-uORF1 target site. **D:** Schematic representation of the editing vector targeting uORF3. The red nucleotide sequence at the scissor icon marked the sgRNA-uORF3 target site. **E:** Sanger sequencing results of the edited uORF3 region. The inserted sequence was highlighted in cyan. The newly introduced premature stop codon was marked in red box. The original uORF3 was indicated by an orange directional indicator underneath.

*CCM_08965* and *CCM_08966* are key genes within the γ-aminobutyric acid (GABA) biosynthetic gene cluster in *C. militaris* (Fig. 4A). To explore post-transcriptional regulatory mechanisms that could be leveraged to enhance GABA production, uORFs located within the 5′ untranslated region (5′ UTR) of *CCM_08965* were selected for targeted editing. In silico promoter analysis of the 1865 bp region upstream of the *CCM_08965* translation start site (ATG) identified two putative transcription start sites (TSSs) (Fig. 4B). RT-PCR analysis using primers positioned at −79 bp (F1), −342 bp (F2), and −609 bp (F3) relative to the ATG paired with a common reverse primer (R) situated within the second exon of *CCM_08965* revealed cDNA-specific amplification for primer pairs F1/R and F2/R. Gel extraction and sequencing of the cDNA-derived amplification products confirmed corrected splicing of the first intron, indicating that the 5′ UTR extends at least 342 bp upstream of the translation start site (Fig. S3). This transcribed 5′UTR region was subsequently analyzed for putative uORFs in the sense orientation. The ORFs were predicted using multiple online tools, including NCBI ORFfinder (https://0-www-ncbi-nlm-nih-gov.brum.beds.ac.uk/orffinder/), NovoPro (https://www.novopro.cn/tools/orf_find.html), and uORF-light (http://www.rnairport.com:443/). The intersection of the predicted results was subsequently considered as the final ORF candidates. Two candidate uORFs, including uORF1 (Fig. 4B, highlighted in blue) and uORF3 (Fig. 4B, highlighted in orange), were identified. uORF1 initiates with a canonical ATG and terminates with TGA, encoding 92 amino acids over 279 bp, while uORF3 is a 63 bp element initiating from a non-canonical CTG start codon (positions −262 to −199) and predicted to encode a 20-aa peptide (Fig. S4).

sgRNAs were designed to target sequences immediately adjacent to the start codons of each uORF (depicted in yellow, Fig. S4A), positioning the PAM to induce DSB proximal to the initiation sites (Fig. 4C and D). The corresponding editing constructs were subsequently introduced into *C. militaris* protoplasts. Repeated attempts to disrupt uORF1 did not yield detectable edited strains. In contrast, transformation with the uORF3-targeting vector yielded several edited strains. Sequence analysis of a representative mutant revealed the insertion of a short nucleotide sequence located 10 bp downstream of the uORF3 start codon, which resulted in a frameshift and introduced a premature termination codon, thereby abolishing uORF3 function (Fig. 4E). These results demonstrated that OmniEdit platform enabled precise CRISPR/Cas9-mediated disruption of regulatory uORFs in *C. militaris*.

To further evaluate the functional consequence of uORF3 editing, we quantified the metabolite GABA in both the WT and uORF3-edited strains. Our results showed that the GABA level in the edited strains was slightly higher than that in the WT. However, this increase did not reach statistical significance (*P* > 0.05). (Fig. S4B and S4C).

### 3.5 One-step multiplexed gene knockout

**Fig. 5:**
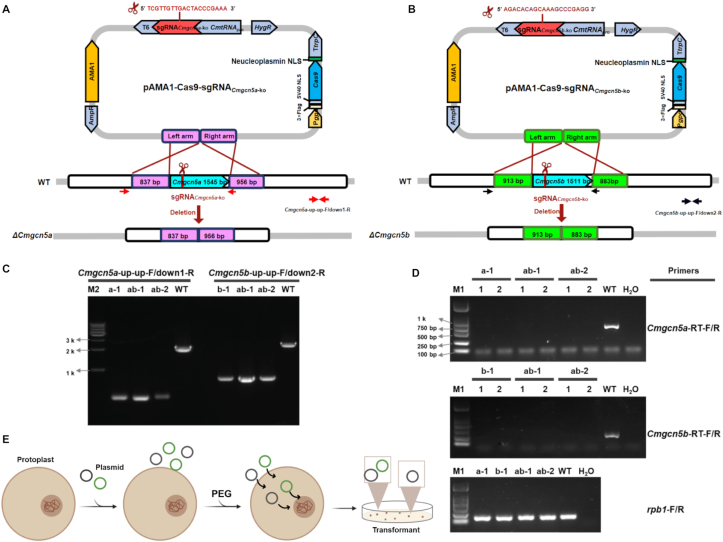
**PEG-mediated co-transformation enables one-step multiplexed gene knockout in *Cordyceps militaris.*** **A:** Schematic overview of the CRISPR/Cas9 strategy used for *Cmgcn5a* gene knockout; **B:** Schematic overview of the CRISPR/Cas9 strategy used for *Cmgcn5b* gene knockout; The left and right arms were donor templates. Arrows indicate the positions of the validation primers. **C:** PCR verification of knockout transformants. M2: 10 kb DNA marker. a-1: *ΔCmgcn5a* single-gene knockout mutant. b-1: *ΔCmgcn5b* single-gene knockout mutant. ab-1 and ab-2: *ΔCmgcn5ab* double gene knockout mutants. WT: Wild-type strain CGMCC 3.16323. **D:** RT-PCR analysis of gene transcription levels in transformants, with *rpb1* as the internal reference gene. Numbers 1 and 2 indicate replicates. **E:** Schematic illustration of PEG-mediated vector co-transformation of *C*. *militaris* protoplasts. Individual plasmids were distinguished by color.

The edible fungus *C. militaris* encodes two distinct GCN5-type histone acetyltransferases, CmGCN5a and CmGCN5b. To enable functional analysis of potential redundancy or cooperation between these two enzymes, a one-step multiplexed knockout strategy was established using the OmniEdit platform. Two gene-specific knockout vectors targeting *Cmgcn5a* and *Cmgcn5b* were independently constructed using an identical CRISPR/Cas9-based design (Fig. 5A and B), each carrying a single sgRNA expression cassette and homology arms flanking the corresponding open reading frame. The two validated knockout vectors were co-transformed at a 1:1 M ratio into WT strain via PEG-mediated protoplast-mediated transformation. PCR screening of hygromycin-resistant transformants identified mutants in which both *Cmgcn5a* and *Cmgcn5b* loci were simultaneously disrupted, yielding a *ΔCmgcn5ab* double-knockout mutant (Fig. 5C and D). Across independent experiments, this co-transformation strategy achieved an average knockout efficiency of approximately 30% (Fig. 5E).

These results demonstrated that the OmniEdit platform enabled efficient, one-step multiplexed gene knockout in *C. militaris* through the co-transformation of independent CRISPR/Cas9 vectors.

## 4 Discussion

This study established OmniEdit as a unified CRISPR/Cas9-based genome-editing framework for *C. militaris*, overcoming a long-standing bottleneck in functional genetics and metabolic reconstruction in mushroom-forming fungi. By integrating diverse genome-editing operations into a single standardized workflow, OmniEdit enables flexible yet precise manipulation of genes, regulatory elements, and metabolic loci, ranging from point mutations, protein tagging, large-fragment deletions, targeted disruption of a regulatory uORF, and multiplex gene knockouts. This unified design reduces reliance on task-specific editing pipelines and provides a scalable platform for systematic dissection of gene function and secondary metabolism in *C. militaris* and related fungal species.

The first successful CRISPR/Cas9-mediated precision point mutations (K9R and K9Q) in a mushroom-forming fungus were reported here, achieved with an average efficiency of ∼4–5% (Fig. 1). A key technical contribution was the implementation of a streamlined screening strategy, in which a short downstream homologous region was deliberately excluded from the repair template (Fig. 1), enabling rapid enrichment of correctly edited transformants and substantially reducing the sequencing burden. Notably, reducing the distance between the Cas9-induced DSB and the intended mutation from 112 bp to 56 bp did not lead to a significant improvement in editing efficiency. This observation contrasts with distance-dependent effects reported in some other systems [29]. One possible explanation is that the reduction in distance achieved here (56 bp) did not reach a critical threshold required to substantially alter repair outcomes [28]. In addition, HDR efficiency in *C. militaris* may be constrained by factors beyond cleavage proximity. Specifically, the extremely high sequence identity between the donor and the endogenous locus, differing by only a single nucleotide, may intrinsically limit strand invasion and recombination efficiency.

The current evaluation of point mutations using the OmniEdit system is largely restricted to the *CmH3* locus. Given that histone genes are typically located in transcriptionally active, open chromatin regions [30] and that CRISPR/Cas9 activity can be affected by local chromatin context [31], the generalizability of these results remains limited. Broader validation across diverse genomic loci will be necessary to fully assess the performance of the system. Nevertheless, the ability to introduce defined point mutations using a unified workflow demonstrates the utility of OmniEdit and supports its potential for systematic protein engineering and functional genomics applications in *C. militaris*.

Fluorescent protein tagging is a foundational approach for visualizing protein localization, and dynamics in living cells and subsequent antibody-based protein assays [32]. Using OmniEdit platform, we established two complementary strategies for C-terminal mCherry tagging of CmGCN5a. *In situ* tagging preserved native regulation control but resulted in weak fluorescence, underscoring the common constraint imposed by low native-promoter activity. In contrast, safe-harbor integration driven by a strong promoter yielded robust and stable fluorescence, enabling clear visualization of CmGCN5a localization (Fig. 2). While strong promoter-driven expression offers clear advantages for imaging, potential effects on protein abundance and localization should be considered [33].

Given that HDR efficiency can be influenced by the length and sequence composition of homology arms [34,35], this study focused on establishing a proof-of-concept for the tagging strategy. Future studies exploring donor templates with varied homology arm designs may further enhance editing efficiency. Notably, the ability to flexibly choose between native-locus and safe-harbor tagging underscores the versatility of OmniEdit for protein localization and downstream functional studies in mushroom-forming fungi. A key finding of this work is that a single Cas9-induced DSB, coupled with an HDR donor, is sufficient to mediate efficient (∼10%) and precise deletion of a large (∼26 kb) BGC in *C. militaris* (Fig. 3). This challenges the conventional paradigm that large deletions necessitate two concurrent cuts to excise the intervening fragment [21] and demonstrates that, under donor-driven HDR conditions, a single-cut strategy can achieve comparable efficiency. By simplifying vector design and reducing the number of required sgRNAs, this “single cut and replace” approach lowers technical complexity while maintaining precision, providing a streamlined strategy for functional interrogation of secondary metabolism and large genomic regions in fungi. This finding is based on a single genomic site, and the efficiency of large-fragment deletion may be influenced by locus-specific factors such as chromatin accessibility, local genomic architecture, and DNA repair pathway preference [[36], [37], [38]]. Therefore, whether this strategy is equally effective at other structurally distinct genomic regions remains to be determined. Systematic validation across multiple loci will be required to establish its broader applicability.

In this study, uORF3 within the 5′UTR of *CCM_08965* was successfully disrupted via a CRISPR/Cas9-mediated genome editing, resulting in a frameshift mutation (Fig. 4). This represents, to our knowledge, the first demonstration of targeted uORF editing in a mushroom-forming fungus. Editing uORFs provides a distinct regulatory strategy compared with gene knockout or promoter engineering, as it enables modulation of translational efficiency without altering the underlying coding sequence or transcript abundance. In plant systems, uORF editing has emerged as an effective approach for fine-tuning gene expression and improving agronomic traits, including stress tolerance and disease resistance [[39], [40], [41]]. Our results indicate that a similar post-transcriptional regulatory layer can be experimentally accessed in mushroom-forming fungi.

Although the edited strains exhibited a slight increase in GABA accumulation compared to the WT, the difference was not statistically significant. This may reflect the tight regulation of GABA homeostasis within fungal metabolism, potentially mediated by compensatory pathways such as the GABA shunt or alternative transamination routes [42], which could buffer the immediate effects of uORF-mediated translational derepression. CCM_08965 protein expression was not assessed in this study, as neither a specific antibody nor a fluorescent fusion construct is currently available, precluding direct evaluation of translational effects.

A notable observation is the difference in editability between two uORFs within *CCM_08965*. Despite repeated attempts, disruption of uORF1 failed, whereas uORF3 editing was achieved, albeit with low efficiency. This discrepancy, together with the overall low editing efficiency of uORF3, underscores the complexity of uORF-targeted editing. Multiple factors may contribute, including chromatin accessibility [43], sequence-dependent features such as genomic context and sgRNA structure [44], and RNA secondary structure within the 5’ UTR [45]. In addition, edited events could not be reliably distinguished by agarose gel electrophoresis and required Sanger sequencing for validation, preventing accurate quantification of efficiency.

These findings highlight current technical limitations in uORF editing in this fungus. Further optimization of editing strategies, including improved sgRNA design, alternative nuclease systems (e.g., Cas12) [46], and direct protein-level or proteomic analyses, will be important for improving efficiency and elucidating the functional impact of uORF regulation. Notably, Cas12 nucleases may facilitate more efficient disruption of *cis*-regulatory elements due to their distinct cleavage properties, as demonstrated in rice [47], and may represent a promising direction for future applications in fungi.

The co-transformation of two independent knockout vectors enabled the simultaneous disruption of *Cmgcn5a* and *Cmgcn5b* with an efficiency of approximately 30% (Fig. 5). This efficiency represents a substantial improvement over traditional methods requiring sequential rounds of screening and is comparable to advanced multiplexing strategies in other fungi [48,49]. Current multiplex genome-editing strategies in models like *Aspergillus nidulans* rely on single-vector systems harboring polycistronic tRNA-gRNA arrays [49]. While effective, these designs increase cloning complexity and reduce modular flexibility. In contrast, our OmniEdit co-transformation strategy allows direct combination of pre-validated single-gene knockout vectors, eliminating the need to assemble complex multi-sgRNA cassettes. Comparable co-transformation strategies have been explored in *Trichoderma reesei* [50], where simultaneous targeting of *vib1* and *lae1* resulted in dual-locus recombination frequencies of 16%. The markedly higher efficiency observed here suggests that OmniEdit provides a more robust and scalable framework for multiplex genome editing in mushroom-forming fungi. These results highlight the utility of OmniEdit for rapid dissection of gene redundancy and genetic interactions, facilitating functional genomics studies that require simultaneous manipulation of multiple loci.

While CRISPR/Cas9 has ushered in a transformative era for gene editing, concerns regarding off-target effects and unintended foreign DNA integration may limit its application in mushroom breeding [51]. A key limitation of the present study is the absence of systematic off-target analysis. Our previous work using an analogous AMA1-based episomal CRISPR/Cas9 system in *C. militaris* provided some preliminary evidence of specificity [8,12]. Whole-genome sequencing of double-gene knockout or knock-in transformants revealed no integration of vector backbone sequences and targeted PCR screening of computationally predicted off-target sites (Cas-OFFinder) detected no off-target mutations [8,12]. However, these results do not substitute for a comprehensive off-target evaluation of the diverse editing strategies employed here, as different sgRNAs and editing contexts may exhibit distinct off-target profiles. To address this limitation, future work will incorporate systematic off-target assessment, including in silico prediction of potential off-target sites for key sgRNAs, followed by experimental validation (e.g., targeted deep sequencing or whole-genome sequencing). Such analyses will be essential to define the specificity of the OmniEdit system and ensure its reliability for industrial applications.

Although the OmniEdit system was established in *C. militaris*, preliminary experiments in the closely related species *Cordyceps chanhua* using the same AMA1-based plasmid system achieved successful gene editing (data not shown), suggesting potential applicability across the *Cordyceps* genus. Nevertheless, species-specific factors such as transformation efficiency, DNA repair pathway preferences, and target site accessibility may influence editing outcomes, and systematic evaluation across a broader range of fungal species will be required to fully establish the generalizability of this platform.

Most reported genome editing systems in macrofungi rely on a single strategy and, in many species, are limited to establishing the system, often targeting only auxotrophic marker genes [52]. These approaches typically employ NHEJ-mediated disruption or simple knock-ins, [53]. In species such as *Ganoderma lucidum* [54], ribonucleoprotein (RNP)-based knockouts have been reported but the lack of effective selection markersgreatly increases screening burden [52]. To our knowledge, the OmniEdit system represents the first integrated platform enabling multiplex gene editing in edible mushrooms. Unlike previous reports that primarily focused on single-gene modifications in macrofungi [53], our system unifies diverse editing strategies such as point mutation, *in situ* tagging, and large fragment deletion within a single CRISPR/Cas9-based framework.

## 5 Conclusion

In summary, OmniEdit shifts the paradigm from simple gene disruption to precise genomic architecture and metabolic reconstruction in mushroom-forming fungi. As a standardized and modular framework, it provides a powerful toolkit for developing *C. militaris* into a high-performance microbial cell factory. This platform not only accelerates industrial strain optimization but also serves as a valuable blueprint for advancing fungal biotechnology across diverse species.

## CRediT authorship contribution statement

**Mengqian Liu:** Writing – review & editing, Writing – original draft, Methodology, Investigation. **Sijin Chen:** Methodology, Investigation. **Jingting Wang:** Investigation, Data curation. **Guoliang Meng:** Methodology, Investigation. **Caihong Dong:** Writing – review & editing, Supervision, Funding acquisition, Conceptualization.

## Data availability statement

All relevant data are available from the corresponding author upon reasonable request.

## Declaration of competing interest

The authors declare that they have no known competing financial interests or personal relationships that could have appeared to influence the work reported in this paper.
