# Supplementary material for: OmniEdit: A unified CRISPR/Cas9 platform for precise genome engineering and strain optimization of the Cordyceps militaris cell factory
Source: Synth Syst Biotechnol. 2026 May 17;14:255–66. doi: 10.1016/j.synbio.2026.04.015 (PMC13197704; doi:10.1016/j.synbio.2026.04.015)
Supplement: Multimedia component 1 [file mmc1.docx]

**Supplementary material**

**Fig. S1**

**A**


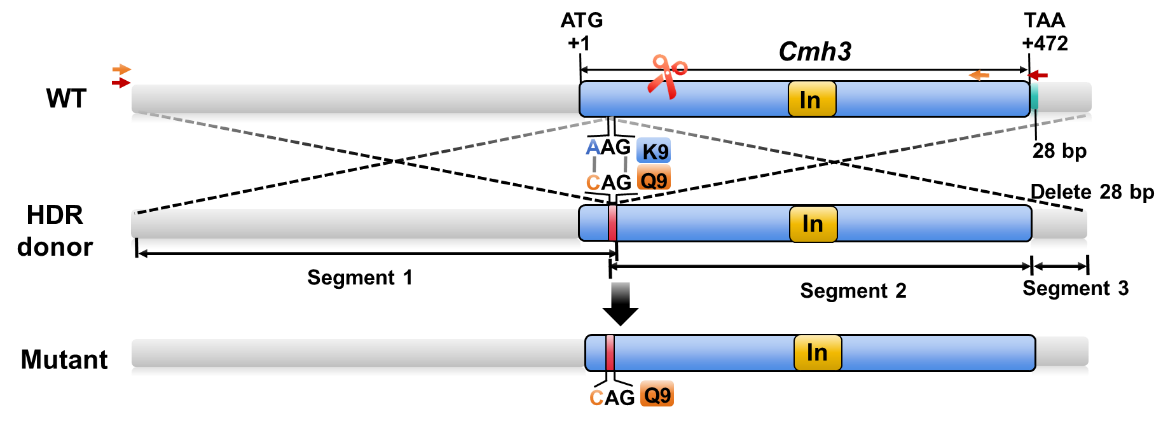


**B**


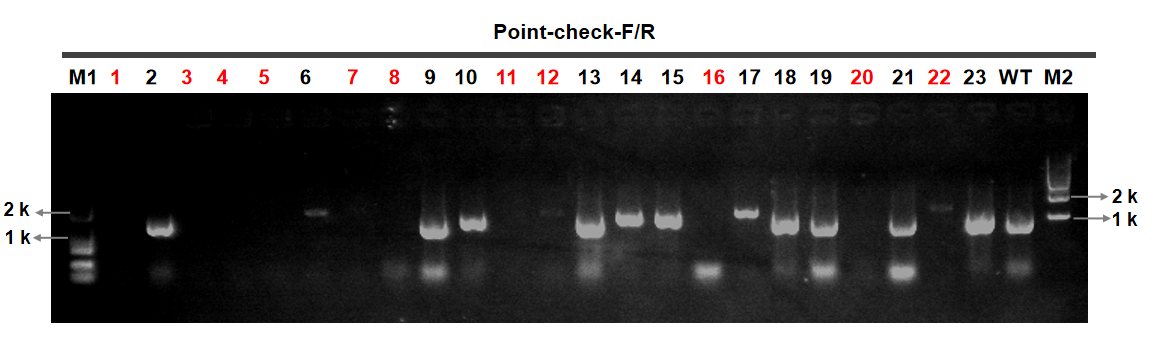


**C**


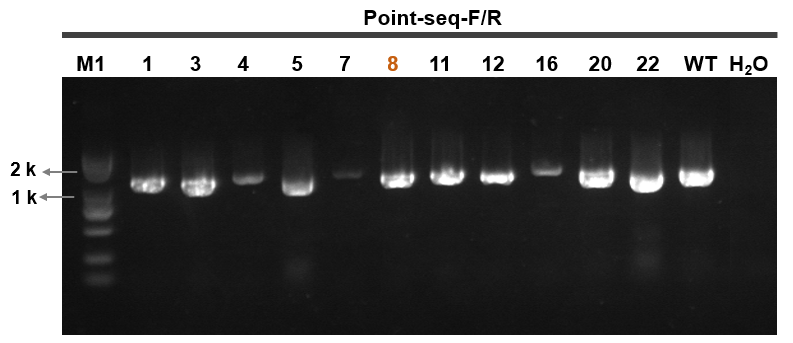


Fig. S1 OmniEdit-mediated point mutation strategy and validation of K-to-Q substitution

**A:** Schematic overview of the point mutation editing strategy. The scissors icon indicated the sgRNA target site. Segments 1, 2, and 3 represented the three fragments amplified for vector construction. Segment 3 was intentionally amplified without the 28 bp region immediately downstream of the stop codon (highlighted in green) to facilitate subsequent screening. Primers used for primary screening (point-check-F/R) were marked in red, and those for secondary sequencing validation were shown in orange-yellow. “In” denoted gene introns. **B:** Primary PCR screening of transformants. Samples that failed to yield an amplification product were labeled with red numbers and were subjected to a second PCR validation. M1: 2 kb DNA marker. WT: Wild-type strain CGMCC 3.16323. **C:** Secondary PCR verification of transformants. PCR products were subsequently subjected to Sanger sequencing. Samples highlighted in orange corresponded to transformants confirmed as positive by sequencing.

**Fig. S2**

**A**

**
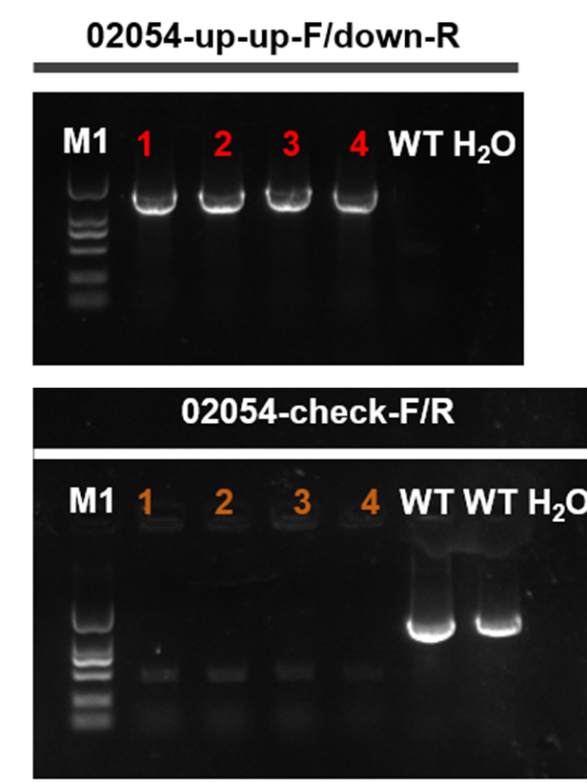
**

**B**

**
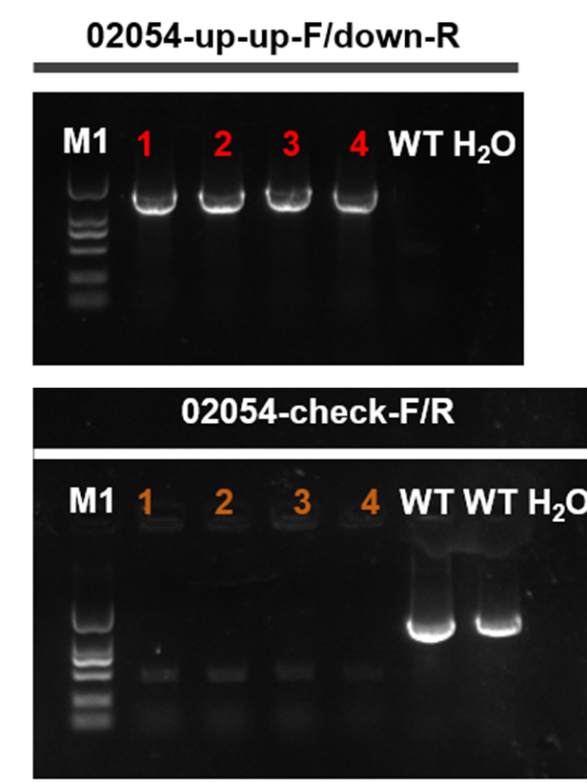
**

Fig. S2 PCR verification of transformants

**A:** Primary PCR screening of transformants (lower panel). Samples that failed to produce an amplification product were labeled with red numbers and were subjected to second PCR validation. M1: 2 kb DNA marker. WT: Wild-type strain CGMCC 3.16323. **B:** Secondary PCR verification of transformants (upper panel). The resulting products were sent for Sanger sequencing. Samples highlighted in orange corresponded to positive transformants confirmed by sequencing.

**Fig. S3**

**A**


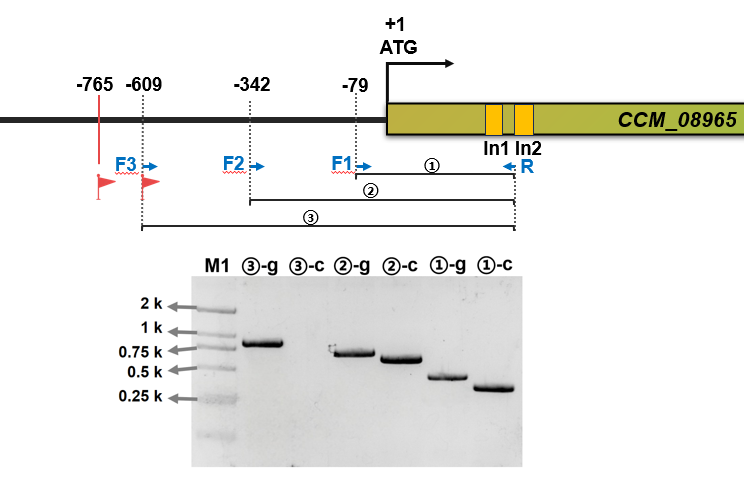


**B**


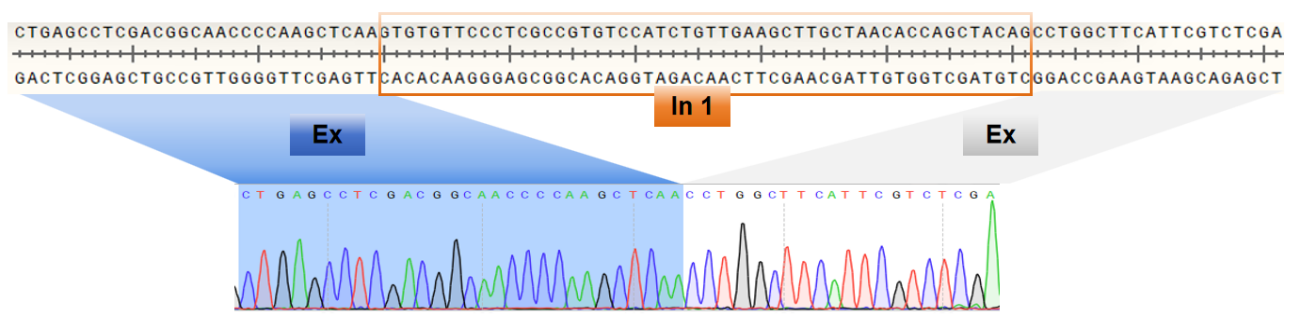


Fig. S3 Identification of the 5’ UTR of *CCM_08965*

**A:** Determination of the 5’ UTR length of *CCM_08965* by RT-PCR. Agarose gel electrophoresis showing the amplified 5’ UTR region from *C. militaris* cDNA. All primers were indicated by blue arrows. Amplified products (numbered 1–3 with circles) and introns of different lengths (labeled as “In”) were marked. The lowercase letters “g” and “c” denote genomic DNA and cDNA, respectively. **B:** Sequencing of the cDNA products confirmed the amplification of intron-spanning regions. “Ex” indicated exons, and “In” indicated introns.

**Fig. S4**

**A**


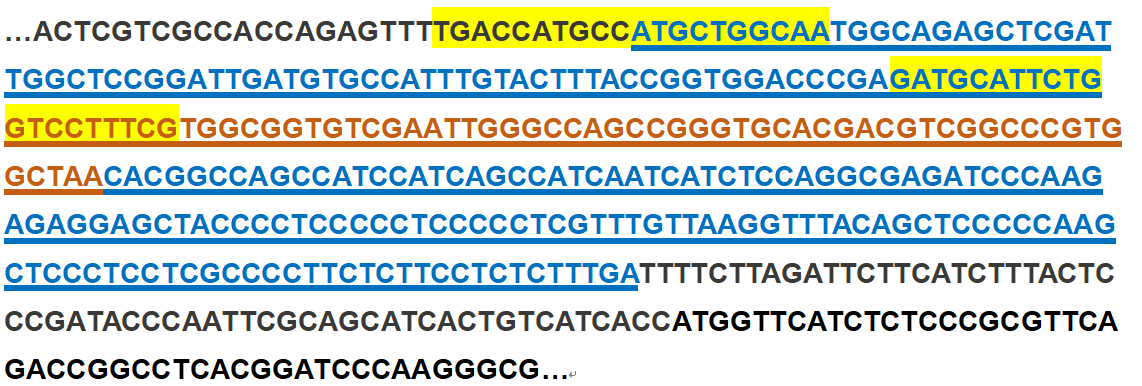


**B**


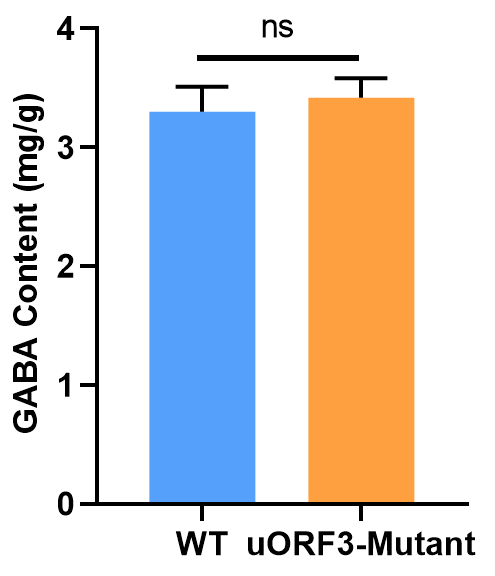


**C**


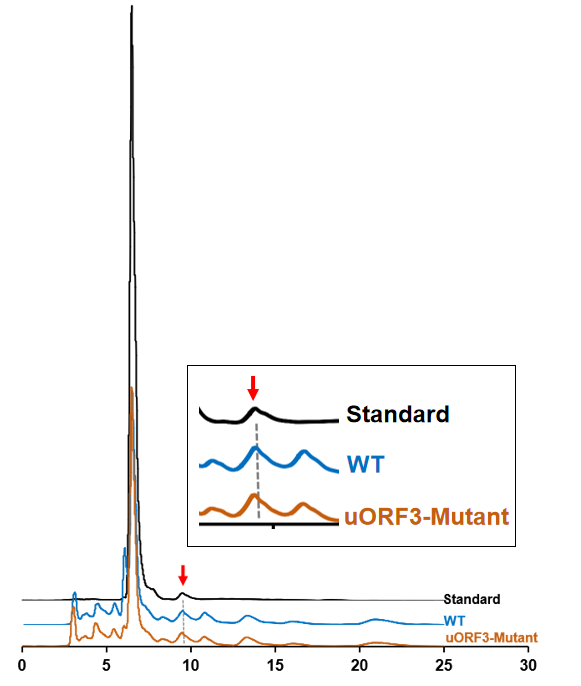


Fig. S4 Targeting strategy for uORF1 and uORF3 and GABA detection chromatograms

**A:** uORF sequence and sgRNA targets. The nucleotide sequence of uORF1 was displayed. The binding sites for the specific sgRNAs designed to edit uORF1 and uORF3 are highlighted in yellow. **B**: Comparison of GABA content between WT and uORF3 mutant. Data represented three biological replicates. Statistical significance was assessed by Student’s *t*-test; “ns” indicated no significant difference. **C:** GABA detection chromatograms (265 nm) of WT (blue) and the uORF3 mutant (orange); The standard is shown in black. The red arrow indicates the GABA peak.

**Table S1 Primers used in this study**

| **Primers** | **Sequence (5’–3’)** | **Function** |
| --- | --- | --- |
| F1 | ATAAATGCTTCAATAATATTTTCGAGATAAGTACAGGGTA | Constructing homologous template DNA in point mutation (H3R9) vectors |
| R1 | CACCAGTGGACCTTCTAGCGGTTTGCTTGGTGCGAGCCAT |  |
| F2 | CGCTAGAAGGTCCACTGGTGGCAAGGCTCCTCGCAAGCAG |  |
| R2 | GTCGTATCAAAGCAATGTCATTAGTTGCGCTCACCGCGGA |  |
| H3-down-F | TGACATTGCTTTGATACGACGGGAAACATGGATCTTTAGG |  |
| H3-down-R | ACTCTTCCTTTTTCAATATTTGACAAGTAGTTTCCTATCG |  |
| Bste-5srRNA-F | GAATGATCCGCCTAAAGCGTGGTCACCACATACGACCATACCCACTGGAA | Constructing the sgRNA1 expression cassette in point mutation vectors |
| 5S-sgRNA1-R | CAACACAACACTCTCAATATAACATACAACACCAGGGATTCGC |  |
| g-sgRNA1-F | CGACGGTACCAGGCTTATAAGTTTTAGAGCTAGAAATAGCAAGT |  |
| Bste-gRNA-scaf-R | TCACCGGGCGGCTCGTACAAGGTCACCGCCTGTCAAAAAAGCACCGACTC |  |
| 5S-sgRNA2-R | CAACACAACACTCTCAATATAACATACAACACCAGGGATTCGC | Constructing the sgRNA2 expression cassette in point mutation vectors |
| g-sgRNA2-F | ATATTGAGAGTGTTGTGTTGGTTTTAGAGCTAGAAATAGCAAGT |  |
| Q9-up-R | CACCAGTGGACTGTCTAGCGGTTTGCTTGGTGCGAGCCAT | Constructing homologous template DNA in point mutation (H3Q9) vectors |
| Q9-down-F | CGCTAGACAGTCCACTGGTGGCAAGGCTCCTCGCAAGCAG |  |
| Point-check-F | CGCCACCATCATCAACATGC | Primers for the first round of point mutation transformant screening |
| Point-check-R | ATCTGTGCGCCAGGTGATAC |  |
| Point-seq-F | TGACCGAGCAACCTTAACAA | Primers for the second round of point mutation transformant screening |
| Point-seq-R | AAGACTGGAAGCGGAGATCGC |  |
| V-ssp-hr-check-F | GCATCCGCTTACAGACAAGC | Vector ligation verification primers |
| V-ssp-hr-check-R | GGAATAAGGGCGACACGGAA |  |
| 5S-mcherry-sgRNA-R | CGAAATTGAGGCGCAACAAGAACATACAACACCAGGGATTCGC | Constructing the sgRNA expression cassette in mcherry fusion vector *in situ* |
| g-mcherry-sgRNA2-F | CTTGTTGCGCCTCAATTTCGGTTTTAGAGCTAGAAATAGCAAGT |  |
| 612-mcherry-up-F | ACCCTGATAAATGCTTCAATAATATTCCGGGCGTCATCGAAGAGCG | Constructing homologous template DNA in mcherry fusion vector *in situ* |
| 612-mcherry-up-R2 | GAGCCTCCACCTCCGGATCCCTTCTCTGGCTCCAGATGTG |  |
| 612-mcherry-F | GAAGGGATCCGGAGGTGGAGGCTCCGGCGGAGGCGGTTCC |  |
| 612-mcherry-R | CCTTTCCAAACTTCACTTATCGTCGTCATCCTTG |  |
| 612-cherry-down--F | ATAAGTGAAGTTTGGAAAGGTACCTTCATTCAAG |  |
| 612-cherry-down-R | CATACTCTTCCTTTTTCAATATTTGATGGAGCGAGGTTGGAG |  |
| 198-pro-F | CTGGGATACGGATGAGCTCGGTTTAAACACTTCGTTTTAAATACCGCGC | Constructing homologous template DNA in mcherry fusion vector *in situ* |
| a-p-R | ATCTTCCTTAAGGTCTGACATGGGCTGCGAAAGGAGTTAGCATGTGT |  |
| a-F | CTCCTTTCGCAGCCCATGTCAGACCTTAAGGAAGATAGTATGTTGTCT |  |
| a-R | ctccgccggagcctccacctccGGATCCTTACTTCTCTGGCTCCAGAT |  |
| a-mcherry-F | aggtggaggctccggcggaggcggttccatggtgagcaagggcga |  |
| mcherry-R | CATTCACTTATCGTCGTCATCCTTGTAATCcttgtacagctcgtccatgc |  |
| Mcherry-trpC-F | agGATTACAAGGATGACGACGATAAGTGAATGCCATGCTCCCCATCACC |  |
| 198-TrpC-R | GAGGCATACGTGCCACGTTTAAACGTTGTTGTTCTTGTTGCTTCGGC |  |
| 2054-sgRNA-R | TGTCCACGCAAAAGAACCTTGTTTTAGAGCTAGAAATAGCAAGT | Constructing the sgRNA expression cassette in cluster deletion vector |
| 2054-sgRNA-F | AAGGTTCTTTTGCGTGGACAAACATACAACACCAGGGATTCGC |  |
| 2066-sgRNA-R | TGCCATGGTCGTGCCATGCCGTTTTAGAGCTAGAAATAGCAAGTT |  |
| 2066-sgRNA-F | GGCATGGCACGACCATGGCAAACATACAACACCAGGGATTC |  |
| ustio-up-F | ACCCTGATAAATGCTTCAATAATATTCGCTCGTGAATCCATATGCT | Constructing homologous template DNA in cluster deletion vector |
| ustio-up-R | GTGAGTCCGCCACCTGCGCTACATACAAGCCCGCTA |  |
| ustio-down-F | CTTGTATGTAGCGCAGGTGGCGGACTCACTATG |  |
| ustio-down-R | ACTCATACTCTTCCTTTTTCAATATTTACGTTACAGCGGTGCAGTT |  |
| 8965sgRNAo3-R | CGAAAGGACCAGAATGCATCAACATACAACACCAGGGATTCGC | Constructing the sgRNA expression cassette in uORF3-editing vector |
| 8965sgRNAo3-F | GATGCATTCTGGTCCTTTCGGTTTTAGAGCTAGAAATAGCAAGT |  |
| 8965sgRNAo1-R | TTGCCAGCATGGCATGGTCAAACATACAACACCAGGGATTCGC | Constructing the sgRNA expression cassette in uORF1-editing vector |
| 8965sgRNAo1-F | TGACCATGCCATGCTGGCAAGTTTTAGAGCTAGAAATAGCAAGT |  |
| *Cmgcn5a*-5s-sgRNA-R | TTTCGGGTAGTCAACAACGAGTTTTAGAGCTAGAAATAGC | Constructing the sgRNA expression cassette in *Cmgcn5a*-knockout vector |
| *Cmgcn5a*-sgRNA-scaffold-F | TCGTTGTTGACTACCCGAAAAACATACAACACCAGGGATTC |  |
| *Cmgcn5a*-leftarm-F | GAATGATCCGCCTAAAGCGTGGTCACCCTTATCTGACATGATGCGAGAGTTG | Constructing homologous template DNA in *Cmgcn5a*-knockout vector |
| *Cmgcn5a*-leftarm-R | GCGTGGCTAATGGCAGCGCTTAGTTATCGAAGATGGACTCG |  |
| *Cmgcn5a*-rightarm-F | CGAGTCCATCTTCGATAACTAAGCGCTGCCATTAGCCACGC |  |
| *Cmgcn5a*-rightarm-R | TCACCGGGCGGCTCGTACAAGGTCACCTGATGGAGCGAGGTTGGAGTTC |  |
| *Cmgcn5b*-5s-sgRNA-R | CCTCGGGCTTTGCTGTGTCTAACATACAACACCAGGGATTCGC | Constructing homologous template DNA in *Cmgcn5b*-knockout vector |
| *Cmgcn5b*-sgRNA-scaffold-F | AGACACAGCAAAGCCCGAGGGTTTTAGAGCTAGAAATAGCAAGT |  |
| *Cmgcn5b*-leftarm-F | ACCCTGATAAATGCTTCAATAATATTCTGATCTCCGAACGACAACC | Constructing homologous template DNA in *Cmgcn5b*-knockout vector |
| *Cmgcn5b*-leftarm-R | GGTTTCTCCCACTCACGTGACTGTACGACGCTGAAG |  |
| *Cmgcn5b*-rightarm-F | CGTCGTACAGTCACGTGAGTGGGAGAAACCAGACGG |  |
| *Cmgcn5b*-rightarm-R | ACTCATACTCTTCCTTTTTCAATATTTCATCACGAACCGCAAGAGC |  |
| 07612-up-up-F/  *Cmgcn5a*-up-up-F | GTTGATGCATCGCAGAAGCTG | Primers for mutation *Cmgcn5a*-knockout transformant |
| Down1-R | AAGGTACCTTTCCAAACTCG |  |
| *Cmgcn5b*-up-up-F | CACAAGGTTGTCTTGGCGTG | Primers for the mutation *Cmgcn5b*-knockout transformant |
| Down2-R | TACCCTCCTCCTCCCCATTC |  |
| *Cmgcn5b*-RT-F | CACAAAACACCACACGGTCC | RT-PCR primers for *Cmgcn5b* |
| *Cmgcn5b*-RT-R | CCTCGGGCTTTGCTGTGTCT |  |
| *Cmgcn5a*-RT-F | AGGACAAAGATGGAGCGCAA | RT-PCR primers for *Cmgcn5a* |
| *Cmgcn5a*-RT-R | TTCGCTCTTCGATGACGCCC |  |
| Safe-up-up-F | CTTCAACGTTCTCAACGCAC | Primers for the mutation *Cmgcn5a-mCherry* transformant |
| mcherry-R | AGCCCATGGTCTTCTTCTGC |  |
| gpdA-R | TTCCTTTGAACTGAAGAATG |  |
| 02054-check-F/  02054-up-up-F | CGCTCATAAATTGCGCTCGT | Primers for the mutation cluster-deletion transformant |
| 02054-check-R | AGAAAACGGTCTGGCTGAGG |  |
| down-R | TGCAAGTTGCCGCTTGTTAC |  |
| F1 | CTTCTCTTCCTCTCTTTGAT | Primers for verifying uORF region transcription |
| F2 | AATAATGAAAGAAGACGTGG |  |
| F3 | GCCATGCTCTTGTTACGCTG |  |
| R | ACGCGGGAGAGATGAACCAT |  |
| IN-F | GAGTACAATGACGAGTTTAC |  |
| IN-R | TCGAGACGAATGAAGCCAGG |  |
